# Australian Aboriginal and Torres Strait Islander-focused primary healthcare social and emotional wellbeing research: a systematic review protocol

**DOI:** 10.1186/s13643-015-0180-6

**Published:** 2015-12-30

**Authors:** Sara Farnbach, Anne-Marie Eades, Maree Lisa Hackett

**Affiliations:** The George Institute for Global Health, PO Box M201, Missenden Road, Camperdown, New South Wales Australia; The University of Sydney, Sydney, New South Wales Australia

**Keywords:** Aboriginal and Torres Strait Islander, Indigenous, Australia, Primary healthcare, Research, Social and emotional wellbeing, Mental health

## Abstract

**Background:**

Research with a focus on Aboriginal and Torres Strait Islander Australian’s (hereafter referred to as Indigenous^1^) needs is crucial to ensure culturally appropriate evidence-based strategies are developed to improve health. However, concerns surrounding this research exist, arising from some previous research lacking community consultation, resulting in little community benefit or infringing on important cultural values. *Values and Ethics*: *Guidelines for Ethical conduct in Aboriginal and Torres Strait Islander Health Research* (hereafter referred to as Values and Ethics), developed by The National Health and Medical Research Council of Australia in 2003, is the ethical standard for Indigenous-focused health research. Researchers must address its Values in research design and conduct. However, its impact on research processes is unclear. Local Protocols should also be considered. This review aims to systematically examine practices related to Values and Ethics, Local Protocols and the processes of conducting Indigenous-focused primary healthcare research in collaboration with external researchers.

**Methods:**

The following electronic databases and grey literature will be searched (2003 to current): MEDLINE, EMBASE, CINAHL, Informit and HealthInfoNet—an Indigenous-specific research and program website. Indigenous-focused research will be included. Research must be conducted in one or more primary healthcare services, in collaboration with external researchers and with a focus on social and emotional well being. One reviewer will review titles and abstracts to remove obviously irrelevant research articles. Full-text research articles will be retrieved and independently examined by two reviewers. Data and quality assessment will be completed by one reviewer and verified by a second reviewer. Quality will be assessed using modified versions of established quality assessment tools.

**Discussion:**

This review will provide information on research processes and the impact of Values and Ethics on Indigenous-focused primary healthcare research, informing communities and primary healthcare staff around research practices, and researchers and policy makers of strengths and weaknesses of practice.

**Systematic review registration:**

PROSPERO CRD42015024994

**Electronic supplementary material:**

The online version of this article (doi:10.1186/s13643-015-0180-6) contains supplementary material, which is available to authorized users.

## Background

Health research intended to benefit Aboriginal and Torres Strait Islander (hereafter referred to as Indigenous^1^) people has frequently been conducted poorly, with little collaboration between the researchers and Indigenous communities, often without providing any short- or long-term benefit to the communities or individuals involved. Non-Indigenous researchers have commonly held control of Indigenous-focused research [1], and health research has been criticised for its repetitive portrayal of poor Indigenous health status, lack of community collaboration [2], and little or no clear positive benefit to the communities or individuals involved [3, 4]. These factors, on the historical backdrop of colonisation, have led to a distrust of Western researchers by some Indigenous people [5, 6].

There has been a concerted effort to change the approach to Indigenous-focused health research, placing a greater emphasis on community benefit, collaboration, knowledge transfer and relationships between communities and researchers. This has resulted in the development of several strategies to improve research processes. The *Interim Guideline on Ethical Matters in Aboriginal and Torres Strait Islander Health Research* was developed in 1991 [7]; however, it was quickly revised, as it was found to lack focus on sound research principles [8], failed to establish processes for the ongoing review of projects, and was considered to be ‘watered-down’ from its original principles [5].

In 2003 the revised *Value and Ethics*: *Guideline for Ethical Conduct in Aboriginal and Torres Strait Islander Health Research* [9] (hereafter referred to as Value and Ethics) was published. Values and Ethics [9] was developed as an authoritative statement and has the same status as the National Statement for Health Research [10]. It outlines the following Values that researchers, academic institutions and funders must consider when conducting Indigenous-focused health research: reciprocity, respect, equality, responsibility, survival and protection, and spirit and integrity [9]. The impact of Values and Ethics [9] on research processes, and on community benefit, is unclear [11, 12].

Values and Ethics [9] emphasises several key principles, including the conduct of research that addresses community-determined priority areas, developing community capacity through skills or knowledge development and including communities as equal partners in the research process. To complete research according to these principles, researchers need to be adaptable, and additional time and resources may be necessary in comparison with non-Indigenous focused research. However, there has been a perception that funding agencies, institutional and academic structures rarely allocate sufficient resources or make allowances for researchers to complete this work. This presents a unique and challenging environment for Indigenous-focused research to occur [11–13]. An evaluation of Values and Ethics [9] is being jointly conducted by The Lowitja Institute and National Health and Medical Research Council of Australia and revisions of the document are under consideration [14, 15].

Increasingly, researchers and communities are documenting components of setting up and managing research projects. Examples of community-controlled research [16], community participation in research [17], documentation of Local Protocols [18, 19], a description of important principles for research [20] and recommendations for completing specific research methods with Indigenous communities, for example conducting survey-based research [8], have been published. However, there are few examples specific actions taken by researchers to address Values and Ethics [9] when conducting health research [21, 22].

One setting where consideration of Values and Ethics [9] is required is research conducted in Primary Health Care (PHC) services. PHC is an important component of the healthcare system. Effective PHC in Indigenous communities has been effective in improving patient outcomes and reducing costs in the hospital system [23]. Health research set in, and relevant to Indigenous communities is needed to ensure that services are of high quality, use the best available evidence, and are culturally appropriate. PHC research is a challenging and resource-intensive process [24], and additional challenges exist when conducting research in Indigenous-focused PHC services [25]. PHC research may be initiated externally, by researchers who identify a problem and approach PHC services to participate, or initiated within a PHC service, where staff identify a problem and conduct their own research—they may also invite external researchers to be involved.

Maintaining and improving the social and emotional wellbeing (SEWB) of Indigenous people is often the goal of PHC services and staff. The term SEWB describes a strength-based holistic perspective of mental health that acknowledges the socio-historical and personal influences on mental health [26]. This term is preferred by some communities, including by many Indigenous Australians. The SEWB of Australia’s Indigenous people is poor compared to Australia’s non-Indigenous population. Suicide rates are twice as high, and Indigenous people are nearly three times as likely to experience high or very high levels of psychological distress than the non-Indigenous population [27]. This disparity exists within a complex historical and social environment. Evidence-based strategies to improve the SEWB of Indigenous communities must be developed.

Conducting Indigenous-focused health research in the PHC setting is challenging; however, it is crucial, as culturally appropriate SEWB services are needed to address the disparity in health between Indigenous and non-Indigenous Australians [13]. An understanding of externally and internally initiated PHC-based research to improve SEWB, including the barriers and enablers to conducting research in this setting, is needed.

We will undertake a systematic review of research conducted with collaboration between Australian PHC services and external researchers, and a focus on improving Indigenous SEWB.

## Methods

This protocol has been registered with PROSPERO CRD42015024994 and reported adhering to the *Preferred Reporting Items for Systematic Reviews and Meta*-*Analyses*-P (PRISMA) statement [28]. Research will be assessed according to *Rationale and Standard for the systematic review of qualitative literature in health services research* [29] and *MOOSE Guidelines for Meta*-*Analyses and Systematic Reviews of Observational Studies guidelines* [30].

### Objective

We will systematically review the conduct of published Indigenous-focused SEWB PHC research in relation to Values and Ethics [9]. Our primary aim is to identify actions, (as reported by the author and identified by the reviewers), that relate to Values and Ethics [9] and Local Protocols (any processes or procedures developed by a community that researchers are expected to adhere to when conducting research or interacting with the community). Our secondary aims are to identify the enablers and barriers to research (as reported by authors) and to comment on ways the research may be translated into practice.

This review will support improved community-researcher relationships by providing Indigenous communities and PHC staff with information to understand current practices, and policy makers and researchers working in the field with information on how research is planned and implemented in line with Values and Ethics [9].

#### Types of research

Research using qualitative, quantitative or mixed methods in the PHC setting, designed to improve Indigenous SEWB will be included. For the purpose of this review, this includes research addressing workforce issues, training, service coordination, resource development, evaluation of interventions, PHC planning, service-level policy, services, processes or the evidence base related to PHC. Only research where the researchers generated original data will be included.

Published evidence and grey literature will be included, including journal articles, reports and evaluations completed by external researchers or PHC service staff.

#### Research setting

Research must be mostly conducted (where at least half of the research or recruitment occurs) in one or more PHC services and include collaboration between PHC service staff and external researchers.

#### Types of participants

Eligible research must have an explicit focus on Australian Indigenous patients or staff of an Indigenous-focused PHC service.

#### Types of interventions

Interventions aiming to improve the SEWB of Indigenous people attending PHC services, including those focusing on social, emotional, spiritual and cultural wellbeing will be included. Eligible research will have an explicit focus on one of the following areas:

- The broad concept of SEWB or mental health
- Depression disorders
- Anxiety disorders
- Smoking or alcohol use, including dual diagnosis

#### Excluded research

We will exclude research with no collaboration between external researchers and PHC service staff or patients, e.g., opinion pieces, internal evaluations, resource reviews or literature reviews. Research with a focus on a specific component of SEWB (e.g., violence, suicide, parenting or perinatal care) rather than the broad concept of SEWB will be excluded, with the exception of research related to depression, anxiety, alcohol consumption, smoking and dual diagnosis. Study protocols with no available findings will be excluded.

#### Types of outcome measures

Research meeting the above criteria will be analysed for actions taken that relate to the Values outlined in Values and Ethics [9] and Local Protocols. A list of potential actions, based on Values and Ethics [9], will be used to describe where values were met (or otherwise) according to the definitions in Additional file 1. We will document the Values that have been explicitly followed. Where processes are described that are in line with Values and Ethics [9] but no explicit reference to Values and Ethics [9] is provided, we will describe these actions.

We will outline where researchers have followed Local Protocols. Drawing on the data described above, we will comment on ways the research may be translated into practice and impact on community-researcher relationships.

## Search methods for identification of research

The following databases will be searched (from 2003) to identify research published in English: MEDLINE, EMBASE, CINAHL and Informit. HealthInfoNet, a website containing a regularly updated list of Indigenous-focused health research, programs and other knowledge, will also be searched. This timeframe was selected to correspond with the development of Values and Ethics [9]. A comprehensive search strategy using the following key words will be developed: primary healthcare, Aboriginal and Torres Strait Islander and social and emotional wellbeing. An example of the search strategy is illustrated in Table 1. Programs and projects listed on the HealthInfoNet website, categorised under the social and emotional wellbeing topic area will be reviewed. The full search strategy for other databases will be available upon request.

**Table 1:** Medline via Ovid search strategy

1. (Primary care or General practi* or Primary health care).tw. or Community mental health services/or Family practice/or Home care services/ or Family physicians/or Community health services/or Community health nursing/or Community pharmacy services/or Community health workers/or Preventive health services/
2. (Community mental health* or Family practice or Family medicine or Family physician* or Home care or Home based or Home health* or Community health* or Community nurs* or health visit* or Community pharmac* or Preventive care or Prevention program* or Preventive service* or Preventive health or Health promotion or aboriginal medical service or aboriginal community medical service).tw.
3. 1 or 2
4. oceanic ancestry group/or aborigin*.tw. or indigenous.tw. or torres strait* islander*.tw.
5. exp australia/ or australia*.tw. or au.in. or australia*.in. or northern territory.tw. or northern territory.in. or tasmania.tw. or tasmania.in. or new south wales.tw. or new south wales.in. or victoria.tw. or victoria.in. or queensland.tw. or queensland.in.
6. 4 and 5
7. 3 and 6
8. mental health/
9. mental health.mp.
10. mental disorder.mp.
11. mental disorder/
12. (social adj2 wellbeing).mp.
13. (well adj2 being).mp.
14. (social and emotional wellbeing).mp. [mp=title, abstract, original title, name of substance word, subject heading word, keyword heading word, protocol supplementary concept word, rare disease supplementary concept word, unique identifier]
15. grief/
16. “grief and loss”.mp.
17. Stress, Psychological/
18. stress.mp.
19. (trauma adj2 abuse).mp.
20. domestic violence/or child abuse/or elder abuse/ or spouse abuse/
21. (removal adj2 famil*).mp.
22. substance-related disorders/or alcohol-related disorders/ or amphetamine-related disorders/or inhalant abuse/ or marijuana abuse/or psychoses, substance-induced/
23. (substance adj2 (abuse or misuse)).mp.
24. (family adj2 (breakdown or breakup)).mp.
25. (cultur* adj2 dislocation).mp.
26. Prejudice/or racism.mp.
27. (racial adj2 discrimination).mp.
28. Socioeconomic Factors/
29. (social adj2 disadvantage*).mp.
30. Depression/or depression.mp.
31. anxiety/or anxiet*.mp.
32. distress.mp.
33. 8 or 9 or 10 or 11 or 12 or 13 or 14 or 15 or 16 or 17 or 18 or 19 or 20 or 21 or 22 or 23 or 24 or 25 or 26 or 27 or 28 or 29 or 30 or 31 or 32
32. 7 and 33

Footnote:* includes all available forms of that word e.g Australia* includes Australia, Australians and Australia's

## Data collection and analysis

### Selection of research

All research articles (titles and abstracts) identified during the search will be imported into an EndNote library [31]. Duplicates will be removed. One reviewer (SF) will review titles and abstracts according to the criteria, to remove obviously irrelevant articles. Full text articles will be retrieved, and the remaining articles will be independently examined by two reviewers against the criteria. Disagreement surrounding the inclusion of an article will be resolved by discussion, or reviewed by a third reviewer (MH) if a consensus cannot be reached.

Programs and projects listed on the HealthInfoNet website social and emotional wellbeing page will be reviewed by SF, and obviously irrelevant programs will be excluded. The remaining programs and projects will be reviewed following the process mentioned above. Where there is a lack of clarity surrounding the project or program, up to three attempts will be made to contact the authors, via phone or email, to determine if further documents are publically available. Only programs with a publication, report or evaluation will be included in the review.

### Data management and extraction

Data extraction and quality assessment will be completed simultaneously. Data will be extracted by one reviewer (SF) and verified by a second reviewer (AME). Research articles will be examined and data related to the outcome measures and review questions will be identified and extracted using data extraction forms specificity designed for this review.

To address the primary outcome, actions related to the use of Values and Ethics’ Values (reciprocity, respect, equality, responsibility, survival and protection and spirit and integrity) [9] and Local Protocols as reported by the author or identified by the reviewer will be extracted.

To address the secondary outcomes, the enablers and barriers as reported by the author and the implications for research practice will be extracted.

### Data synthesis

The main findings will include a narrative synthesis of Value and Ethics’ use, enablers and barriers to research, impact on practice and impact on community-researcher relationships.

### Quality assessment of research findings

Research meeting the above criteria will be categorised according to the research method and assessed for quality using the Qualitative Research Checklist from Critical Appraisal Skills Programme [32] (qualitative), Quality Assessment Tool For Quantitative Studies [33] (quantitative), or the Cochrane Collaboration’s tool for assessing risk of bias [34] (clinical trials).

Quality will be assessed by one reviewer (SF) and verified by a second reviewer (AME). Both reviewers will discuss research if a lack of consensus occurs. Assistance from a third interviewer (MH) will be sought if consensus cannot be reached. For research using multiple methods, research will be assessed according to the method that relates most closely to the primary aim of the research. Where mixed methods including a randomised control trial are used, a risk of bias assessment will also be completed. No research will be excluded based on quality.

## Discussion

This systematic review will provide an overview of the research processes, enablers and barriers and impact on practice and on community-researcher relationships, to conducting Indigenous-focused SEWB PHC research in relation to Values and Ethics [9]. The findings from this review will provide Indigenous communities and PHC staff with information regarding current practices, highlight the use of Values and Ethics [9] and enable policy makers and researchers to identify better processes in order to plan and implement future research in line with Values and Ethics [9].

The identification of successful processes will assist future research design. By systematically identifying and collating enablers and barriers encountered when conducting research, this review will fill an important gap in the healthcare literature, relating to the successful and ethical conduct of Indigenous-focused PHC research conducted in collaboration with the external researchers. This review will provide insight into the impact and implementation of Values and Ethics [9].

## Endnotes

^1^ The term “Indigenous peoples” is used throughout the paper refers to all Aboriginal and/or Torres Strait Islander peoples of Australia. It is used to reflect the fact that Australia’s Indigenous people do not represent a homogenous group.

## Additional file

Additional file 1:
**Additional file 1: Definitions and potential actions for the Values. **(32.6 kb)

## Abbreviations

Indigenous: Aboriginal and Torres Strait Islander
Local Protocols: any processes or procedures developed by a community that researchers are expected to adhere to when conducting research or interacting with the community
PHC: primary healthcare
PRISMA: Peferred Items for Systematic Review and Meta-Analysis protocols
PROSPERO: International prospective register of systematic reviews
SEWB: social and emotional wellbeing
Values and Ethics: Value and Ethics: Guideline for Ethical Conduct in Aboriginal and Torres Strait Islander Health Research
