# Supplementary material for: Australian Aboriginal and Torres Strait Islander-focused primary healthcare social and emotional wellbeing research: a systematic review protocol
Source: Syst Rev. 2015 Dec 30;4:189. doi: 10.1186/s13643-015-0180-6 (PMC4697316; doi:10.1186/s13643-015-0180-6)
Supplement: Additional file 1: — Definitions and potential actions for the Values. (32.6 kb) [file 13643_2015_180_MOESM1_ESM.docx]

Appendix 1: Definitions and potential actions for the Values

Potential actions (or processes) related to the Values have been summarised from Values and Ethics [1]. Actions described in articles that appear to meet the criteria will be attributed to the corresponding Value by the reviewers, even if no explicit mention is made of the Values and Ethics document [1].

Some overlap exists between the definitions and actions associated with each Value. Where clear overlap exists we will attribute actions to one Value, for example ‘equitable distribution of benefit’ is associated with reciprocity and equality, however for the purposes of this review actions will be attributed to reciprocity. This list provides some potential actions and examples. It is not an exhaustive list and is not intended to be a checklist for Indigenous-focused health research.

| Reciprocity: Research that demonstrates inclusion, recognises partners’ contributions or ensures that research outcomes include equitable benefits of value to communities and individuals | |
| --- | --- |
| Potential action | Example of potential action |
| Demonstrated intention to contribute to the advancement of the health and wellbeing of participants and community/ies | Implementation of research findings at participating PHCS e.g. incorporation of validated screening tool into medical software |
| Research responding to regional, jurisdictional or international priorities, or community-identified need | A PHCS Research Committee expressing the need for research to develop tailored SEWB strategies for adolescents in their community |
| Nature of the benefit to community/ies, including demonstration of discussions related to benefits prior to approval | Documentation of discussions between researchers and a PHCS Research Committee (prior to approval) concerning the obligations and potential benefits of participation in the research |
| Demonstrated willingness to modify research according to the community/ies values and aspirations | Altering research design from a RCT to a non-randomised design following community concerns about the fairness of randomisation |
| Research processes that enhance capacity of the community/ies beyond the research | Training and involvement of a PHCS staff member with minimal research experience in research design, data collection and analysis to develop the staff member’s skills |
| Respect: Research that acknowledges and affirms the rights of people to have different values, norms and aspirations, is not blind to differences, recognises the contributions of others to the research and its consequences | |
| Potential action | Example of potential action |
| Decision-making processes that acknowledge the diversity of individuals and community/ies | Invitation and involvement of PHCS staff members from several communities as members of the research advisory committee |
| Acknowledgement of the contribution of individuals and communities | Inclusion of PHCS staff members involved in the research as authors on publications related to the research |
| Efforts to minimise the effect of difference blindness^*^ | Adaptation of an app to improve cultural-appropriateness and relevance |
| Incorporation of Indigenous knowledge and experience | Indigenous PHCS staff members guiding the development of a wellbeing program for implementation and evaluation at a PHCS |
| Demonstration of negotiation of agreements about ownership and rights to intellectual and cultural property | Community and researchers jointly conducting research into Traditional Bush Medicine, where an agreement is described that protects and maintains Community ownership of intellectual and cultural property arising from the research |
| Processes of reaching agreements that demonstrate engaging with local values and/or processes | Following PHCS request, researchers present/discuss research idea with the PHCS Research Committee^#^ (local process), and/or modification of the research according to local preference (local values) |
| Community/ies expressed satisfaction with research agreements and processes | Approval from the PHCS Research Committee^#^ for the conduct of a research study at the PHCS |
| Agreements that include data management, publication arrangements and where identity of participants is protected | A contract involving researchers and PHCS that includes: data management, publication arrangement and protection of community and individual identity |
| Equality: Research that recognises the equal value of all people involved in research, distributive fairness and justice | |
| Potential action | Example of potential action |
| Demonstration of equality between individuals, communities and researchers during the research process | Process to gain input from PHCS Research Committee^#^ and/or staff members to ensure interpretation of research findings are correct |
| Demonstration of community/ies’ understanding (and expressed satisfaction) with the research, its potential benefits and their distribution. This includes prior to research the provision of information to the community in a way that is understood and usable | Researchers attending PHCS Research Committee^#^ meeting/s, where all aspects of a potential study are discussed, prior to the provision of consent for the PHCS to participate in the study |
| Responsibility: Research that does no harm to individuals and communities and the things that they value, or processes that ensure researcher accountability to individuals, families and communities | |
| Potential action | Example of potential action |
| Demonstration of researcher transparency including negotiations related to research purpose, methodology, conduct and dissemination of findings | Documentation of upfront communications between researchers and PHCS Research Committee^#^ members related to expected timeframes for the research |
| Establishment of mechanisms to provide ongoing community review of the research | Regular meetings between researchers and PHCS Research Committee^#^ members throughout the research to monitor and review its progress |
| Demonstrated mechanisms to provide feedback to communities related to expressed concerns, values and expectations | Presentation of research findings identified by the community as relevant, at PHCS Research Committee^#^ meetings |
| Agreements related to publications arrangements, joint sign-off or the protection of individual and community identity | Agreements where PHCS staff members and researchers jointly review findings and develop publications |
| Survival and Protection: Research that demonstrates how it will protect personal and collective bonds, or the cultural distinctiveness of communities | |
| Potential action | Example of potential action |
| Demonstration of the contribution to social or cultural bonds among Indigenous families and communities | A research study into maternal health that involves mothers and grandmothers and is conducted with respect to women’s business |
| Existence of safeguards against potential discrimination of individuals or cultures | Decision not to publish certain demographic information (to protect identity) from a study conducted in a small community |
| Contribution to the opportunity for communities to better advocate or enjoy cultural distinctiveness | Identifying a local artist to develop artwork to represent the study, promoting local cultural uniqueness and identity |
| Existence of strategies to reduce/eliminate threats to cultural distinctiveness | Identifying a local cultural mentor to provide cultural advice to researchers to ensure local context is considered |
| Spirit and Integrity: Research that respects the continuity of the past, current and future generations and demonstrates integrity and credibility during the research process. This is an overarching Value that binds the other Values together | |
| Potential action | Example of potential action |
| Demonstration of an understanding of cultural, social and spiritual cohesion, including workable timeframes | Research study timeframes that include reduced/minimal activities for important cultural events, including festivals or celebrations |
| Recognition of the diversity of cultures | Documentation of steps taken by researchers to identify and comply with local protocols for each community in a multi-centre study |
| Demonstration of personnel integrity (specifically during study development) | Researchers learning about local context and priorities by visiting the PHCS several times during study planning and prior to funding application or finalisation of study protocol |
| Commitment to working within the spirit and integrity of the community/ies | PHCS Research Committee^#^ preferences determining appropriate study design and timeframes |
| **Abbreviations:** PHCS – primary healthcare service; RCT – randomised control trial  **Definitions:** ^*^ Difference blindness: to misrecognise of fail to recognise cultural differences. ^#^ PHCS Research Committee: a group that represents and provides oversight of research conducted at a PHCS. This can include community members and/or staff members. | |

**References**

1. National Health and Medical Research Council. *Values and Ethics: Guidelines for Ethical Conduct in Aboriginal and Torres Strait Islander Health Research*. 2003 [1 September 2014]; Available from: <https://www.nhmrc.gov.au/guidelines-publications/e52>.
